# A ‘how‐to’ guide for estimating animal diel activity using hierarchical models

**DOI:** 10.1111/1365-2656.14213

**Published:** 2024-11-19

**Authors:** Fabiola Iannarilli, Brian D. Gerber, John Erb, John R. Fieberg

**Affiliations:** ^1^ Department of Migration Max Planck Institute of Animal Behavior Constance Germany; ^2^ Department of Fisheries, Wildlife and Conservation Biology St. Paul Minnesota USA; ^3^ U.S. Geological Survey, Colorado Cooperative Fish and Wildlife Research Unit and Department of Fish, Wildlife, and Conservation Biology Colorado State University Fort Collins Colorado USA; ^4^ Minnesota Department of Natural Resources Grand Rapids Minnesota USA

**Keywords:** acoustics, camera trapping, circular data, conditional mean, diel activity, hierarchical model, kernel density estimation, marginal mean

## Abstract

1. Animal diel activity patterns can aid understanding of (a) how species behaviourally adapt to anthropogenic and natural disturbances, (b) mechanisms of species co‐existence through temporal partitioning, and (c) community or ecosystem effects of diel activity shifts.
2. Activity patterns often vary spatially, a feature ignored by the kernel density estimators (KDEs) currently used for estimating diel activity. Ignoring this source of heterogeneity may lead to biased estimates of uncertainty and misleading conclusions regarding the drivers of diel activity. Thus, there is a need for more flexible statistical approaches for estimating activity patterns and testing hypotheses regarding their biotic and abiotic drivers.
3. We illustrate how trigonometric terms and cyclic cubic splines combined with hierarchical models can provide a valuable alternative to KDEs. Like KDEs, these models accommodate circular data, but they can also account for site‐to‐site and other sources of variability, correlation amongst repeated measures, and variable sampling effort. They can also more readily quantify and test hypotheses related to the effects of covariates on activity patterns.
4. Through empirical case studies, we illustrate how hierarchical models can quantify changes in activity levels due to seasonality and in response to biotic and abiotic factors (e.g. anthropogenic stressors and co‐occurrence). We also describe frequentist and Bayesian approaches for quantifying site‐specific (conditional) and population‐averaged (marginal) activity patterns.
5. We provide guidelines and tutorials with detailed step‐by‐step instructions for fitting and interpreting hierarchical models applied to time‐stamped data, such as those recorded by camera traps and audio recorders. We conclude that this approach offers a viable, flexible, and effective alternative to KDEs when modelling animal activity patterns.

## Graphical abstract

We illustrate how hierarchical models can be used to estimate animal diel activity whilst accounting for variability in activity levels and sampling effort. These models also allow researchers to more directly evaluate hypotheses regarding how anthropogenic and environmental factors affect animal diel activity.

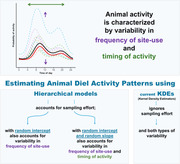

## 1 INTRODUCTION

The time of day in which individuals are typically active is shaped by evolutionary adaptations (e.g. sensory systems), endogenous physiological rhythms, and individual responses to biotic (e.g. intra‐ and interspecific interactions) and environmental factors (e.g. avoidance of unfavourable temperatures: Kronfeld‐Schor et al., 2013). Individuals must weigh the benefits of inactivity (e.g. conserving energy, reducing predation risk) versus the potential fitness gains obtained through vital activities, such as foraging, mating, or patrolling territories. Furthermore, when faced with interspecies competition, individuals may shift their activity patterns, relying on time‐partitioning mechanisms to facilitate species co‐existence in terrestrial and marine environments (Lear et al., 2021).

Animal diel activity patterns—specifically defined through active motion (Hut et al., 2012)—are usually quantified using time‐stamped data gathered via animal‐borne devices, or increasingly, using static sensors, such as camera traps and audio recorders (Desbiez et al., 2021; Wolfson et al., 2023). Historically, activity patterns were described simply using histograms of observed records over the 24‐h cycle (Maffei et al., 2004; Park, 1935), a descriptive approach that ignores diel periodicity in activity. The introduction of non‐parametric circular kernel density estimators (KDEs) was an important step forward, as KDEs accommodate circular data and lead to estimates of activity patterns that meet at the end points (e.g. 00:00 and 23:59 h; Ridout & Linkie, 2009; Rowcliffe et al., 2014). Other modelling approaches that categorize diel activity into states (e.g. day, night, etc.) have also been used (Gallo et al., 2022; Gerber et al., 2024; Rivera et al., 2022; Vallejo‐Vargas et al., 2022), but KDEs are by far the most common approach; Ridout and Linkie (2009), who introduced KDEs as a tool for the analysis of diel activity, has been cited >1430 times as of October 2024 (Google Scholar). Despite their usefulness and ubiquity, current KDEs present several limitations (Table 1).

**TABLE 1:** Modelling and inferential features that can (+) and cannot (−) be accommodated when estimating animal diel activity patterns using current kernel density estimators (KDEs) and hierarchical models. The bottom row lists R packages for implementing these methods.

Features	Current KDEs	Hierarchical models	
		Trigonometric	Cyclic cubic spline
Model circular data to account for the periodic nature of activity patterns	+	+	+
Directly compare activity patterns across groupings of data	−	+	+
Account for sampling effort and estimate probability of activity	−	+	+
Account for repeated observations at the same site	−	+	+
Model variability in frequency of site‐use	−	+	+
Model variability in the timing of activity (i.e. shape of the curves)	−	+	+
Compare across commonly encountered activity patterns (e.g. unimodal, bimodal, etc.)	−	+	−
Directly include covariates	−	+	+
Compare across model structures (e.g. site variability in the frequency of site‐use and/or timing of activity)	−	+	+
Compare relative importance of covariates	−	+	+
R packages	*activity* (Rowcliffe et al., 2014); *overlap* (Meredith & Ridout, 2021; Ridout & Linkie, 2009); *circular* (Agostinelli & Lund, 2017)	*GLMMadaptive* (Rizopoulos, 2020)^a^; *lme4* (Bates et al., 2015)^a^; *glmmTMB* (Brooks et al., 2017)^a^	*mgcv* (Pedersen et al., 2019)^a^
		*brms* (Bürkner, 2021)	

^a^ See Table 2 for details on how to specify random effects in hierarchical models when using these packages.

First, KDEs are typically applied to data that are pooled across multiple spatial locations (sites), assuming independence amongst the observations and ignoring potential site‐to‐site and other sources of variability (e.g. seasonality). This can lead to biased estimators of activity and uncertainty. Second, KDEs can be sensitive to the choice of the bandwidth optimizer, particularly in finite samples or when assumptions of the bandwidth optimizer (e.g. independent observations, normality of the underlying distribution) are not met (Ridout & Linkie, 2009; Rowcliffe et al., 2014). Third, dependence amongst observations is usually only partially addressed. To reduce statistical dependence (which tends to result in wiggly activity patterns, Tutorials 9.3 and 9.4), species' observations recorded a few minutes apart at the same site are typically reduced to a single encounter event (Burton et al., 2015, Iannarilli et al., 2019; Tutorial 9.1). This process reduces within‐site short‐term serial dependence but does not address correlation due to repeated observations at the same site over longer time frames (e.g. days or weeks). Fourth, KDEs cannot be used to estimate hypothesized effects from continuous or categorical covariates. Rather, significance tests are used to explore differences in activity patterns based on categorical groupings of data. Lastly, KDEs currently used to estimate activity patterns only consider records in which an animal was detected, discarding information about when sampling was conducted but the species was not recorded. Thus, these KDEs ignore information about sampling effort which may vary from site to site, estimating only relative activity and making it difficult to compare overall levels of activity across relevant factors (e.g. seasons or experimental groupings of sites). Some limitations of KDEs could be addressed with future developments. For example, methods to accommodate non‐independent data, such as autocorrelated KDEs (AKDEs; Fleming et al., 2015), are available in other contexts but have not yet been adapted for circular data.

Our primary objective in this ‘How‐to’ guide is to illustrate the flexibility and robustness of hierarchical models paired with trigonometric terms or cyclic cubic splines for estimating species activity patterns. These methods are particularly relevant to camera‐trap data but can be applied to any spatially fixed sampling process that records animal activity over time (e.g. audio recorders). In the next sections, we first detail characteristics of activity patterns subject to within‐ and across‐site variation. We then introduce the hierarchical model approaches and use empirical examples to describe how they overcome the limitations of KDEs. We accompany the text with detailed tutorials and code that can be adapted to address common ecological and conservation questions related to animal diel activity patterns.

## 2 METHODS AND RESULTS

### 2.1 Site‐to‐site variability in activity patterns

Animal activity patterns typically vary from site to site due to animals' responses to biotic and abiotic characteristics specific to each site. Site variation is likely to depend on local environmental characteristics (e.g. the extent of canopy cover), the proximity of the sites to important landscape features (e.g. anthropogenic structures, dens or burrows, water), and how species use the sites (e.g. as foraging areas, travel corridors, or rest sites). Furthermore, the presence of a predator, competitor, or dominant conspecific at sites may cause individuals to shift their activity patterns to avoid risky encounters (Guthmann et al., 2023). Temporal processes and species' responses to stressors can also influence the frequency and timing of site‐use (e.g. seasonality: Gallo et al., 2022; Wolfson et al., 2023; human disturbance: Frey et al., 2020; Mayer et al., 2023; artificial light at night: Dominoni & Partecke, 2015). Finally, variations in local population size and in the traits (e.g. sex and age) of nearby individuals could also affect how often and when sites are used.

**FIGURE 1:**
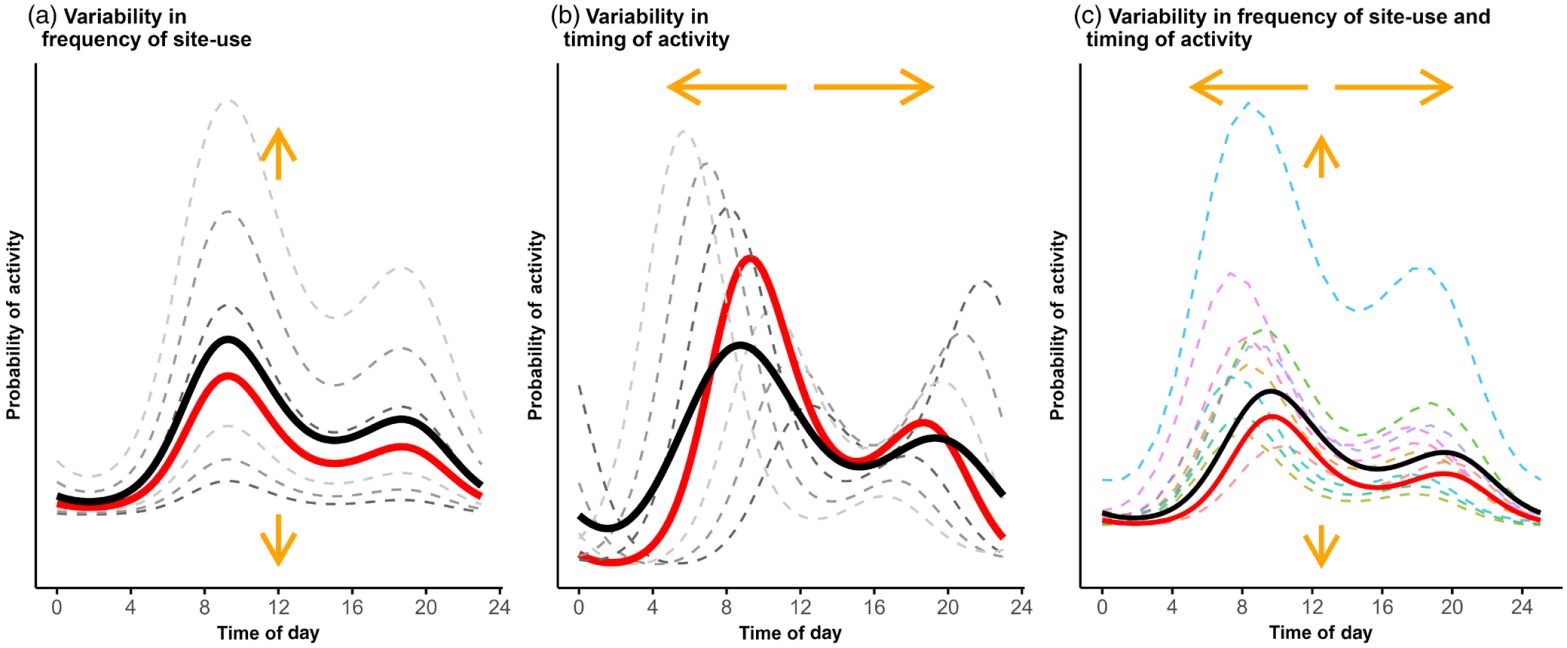
Simulated activity patterns demonstrating variability in the frequency of site‐use (a), timing of activity (b), and their combination (c). The conditional means (red curves) describe activity patterns at a ‘typical site’ (i.e. a site with all random effects equal to 0: Fieberg et al., 2009), whereas the marginal means (black curves) describe activity patterns averaged across the population of sites (‘the population average’, see Box 1). Orange arrows describe the direction of the shifts in activity across sites. Light to dark grey dashed lines represent conditional mean activity patterns at particular sites which have incremental levels of variation in site‐use (a: $\;\tau_{i}=\left[\pm 0.5;\pm 1;\pm 1.5\right],\gamma_{i}=0$) or time of activity (b: $\tau_{i}=0,\;\gamma_{i}=\left[\pm 0.5;\pm 1;\pm 1.5\right]$). In (c), examples of site‐specific activity patterns (dashed, pastel‐coloured curves) were obtained by simulating data using Equation 1 with $\sigma_{\tau }=1$ and $\sigma_{\gamma }=0.3$. Plots were created as described in Tutorials 2 (panels a and b) and 3.1 (panel c).

**FIGURE 2:**
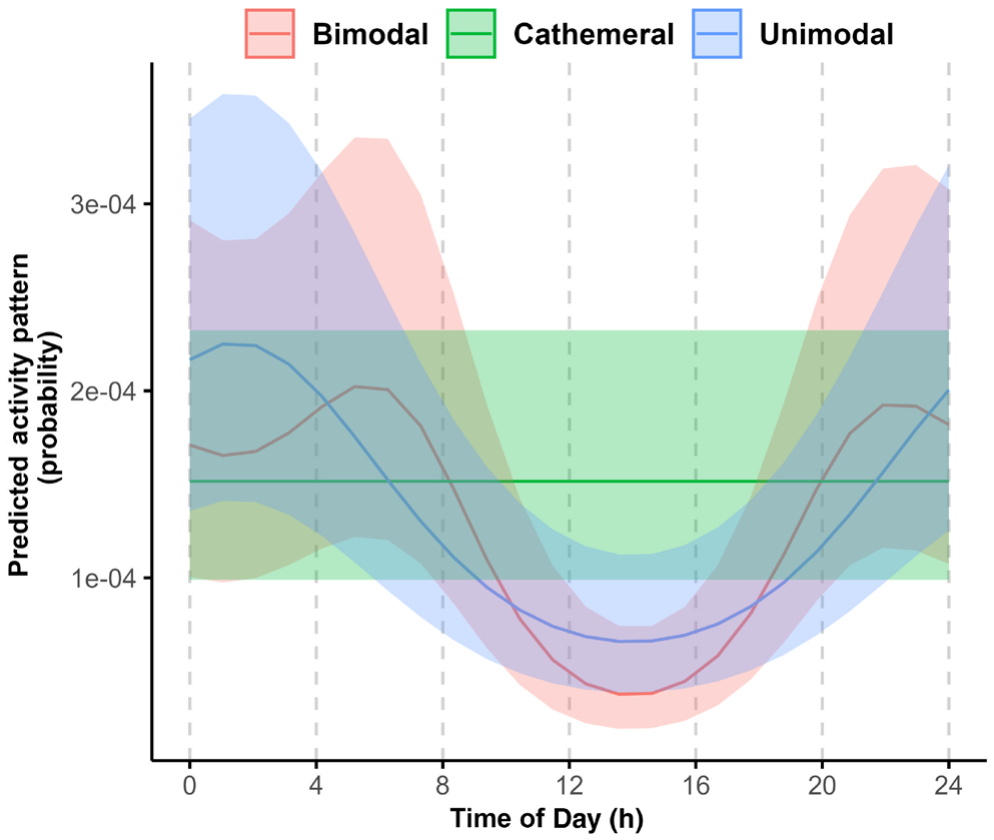
Conditional mean diel activity patterns of coyotes (*Canis latrans*) were predicted based on trigonometric hierarchical models describing cathemeral, unimodal, and bimodal patterns. The bimodal model was most supported. Data were collected in northern Minnesota in 2016–2018 using camera traps.


We can formally decompose site‐to‐site variability in activity patterns into two fundamental components: (1) variability in the overall frequency of site‐use (Figure 1a) and (2) variability in the shape of the activity curve (Figure 1b). We illustrate these two forms of variability separately (Figure 1a,b) and simultaneously (Figure 1c) using simulations based on a hierarchical trigonometric model. Assuming a bimodal activity pattern (i.e. two peaks in activity within the 24‐h cycle), the probability of a species being active at site *i* and at time interval *t*, $p_{\mathrm{it}}$, can be modelled as,

$$y_{\mathrm{it}}\sim \text{Bernoulli}\left(p_{\mathrm{it}}\right)$$

(1) $$\text{logit}\left(p_{\mathrm{it}}\right)=\beta_{0}+\beta_{1}\cos\left(\frac{2\mathrm{\pi t}}{\omega_{1}}+\left(\theta_{0}+\gamma_{i}\right)\right)+\beta_{2}\cos\left(\frac{2\mathrm{\pi t}}{\omega_{2}}+\left(\theta_{1}+\gamma_{i}\right)\right)+\tau_{i}$$

$$\tau_{i}\sim N\left(0,\sigma_{\tau }\right)$$

$$\;\gamma_{i}\sim N\left(0,\sigma_{\gamma }\right)$$

where *y*_*it*_ is 1 when a species is detected as active at site *i* and at time interval *t* (and 0 otherwise), $\beta_{0}$ is a common intercept, $\beta_{1}$ and $\beta_{2}$ describe the amplitude of the two cosine terms, and *t* indicates the time interval within the 24‐h cycle and can be expressed as seconds, minutes or hours (or radians). The terms $\frac{2\mathrm{\pi t}}{\omega_{1}}$ and $\frac{2\mathrm{\pi t}}{\omega_{2}}$ determine the frequency of the two sinusoidal waves (once every 24 and 12 h, respectively when *t* is expressed as hours and $\omega_{1}=24,\omega_{2}=12$). The parameters $\theta_{0}$ and $\theta_{1}$ are phaseshift parameters that control when the peak of each cosine term occurs, and $\gamma_{i}$ are random effects that allow the phaseshift parameters to vary by site (Figure 1b). The parameter $\tau_{i}$ specifies a site‐specific random effect that shifts the two sinusoidal waves vertically (Figure 1a). The random effects, $\gamma_{i}$ and $\tau_{i}$, are assumed to follow normal distributions with mean = 0 and standard deviations equal to $\sigma_{\gamma }$ and $\sigma_{\tau }$, respectively. Hierarchical models allow ecologists to estimate both conditional (i.e. site‐specific) and marginal (i.e. population level) mean activity patterns (Box 1; Fieberg et al., 2009); this choice should depend on one's research questions.

BOX 1 **Site‐specific (conditional mean) and population‐averaged (marginal mean) activity patterns**

Researchers might be interested in exploring either *conditional means*, which describe the temporal use of particular sites (dashed and red curves in Figure 1) or the *marginal mean*, which describes the average pattern of activity across the population of sites (black curves in Figure 1). These mean‐response patterns are frequently referred to as subject‐specific (or, site‐specific in this case) and population‐averaged patterns, respectively (Fieberg, 2024, Section 19.7–19.8; Fieberg et al., 2009; Muff et al., 2020). There are several conditional means (one for each level of the random effect), but it is common to focus on the conditional mean for a typical site (i.e. one with all the random effects in a hierarchical model set equal to zero; red curves in Figure 1). We quantify the marginal mean by integrating over the distribution of random effects (Tutorials 2 and 8). These conditional and marginal means will differ for models formulated using a non‐linear link function (e.g. logit or log, as is common when analysing binary or count data, respectively).

Ecologists should estimate the conditional mean when they are interested in quantifying changes at the site‐level (e.g. how an increase in human disturbance will impact the activity pattern at a particular site). Conditional inference should typically be limited to drivers that can potentially vary within a site (Fieberg et al., 2009). Marginal means are more appropriate for quantifying differences amongst groups of sites that differ in their characteristics (e.g. sites on trails versus at random locations). Importantly, because KDEs pool data across sites, they produce marginal mean activity patterns (Iannarilli, 2020).

### 2.2 Modelling activity using hierarchical models

#### 2.2.1 Trigonometric generalized linear mixed models

Trigonometric regression models describe periodic patterns using a combination of sine and cosine terms (aka, Fourier series) and can be paired with hierarchical models to accommodate site‐to‐site variability. Equation 1 describes bimodal patterns in activity typical, for example, of species that have peaked activity at dusk and dawn (i.e. crepuscular) and accommodates unequal peaks in activity. When $\beta_{2}=0$, this equation produces a unimodal pattern (only one peak within the 24‐h cycle) typical of diurnal and nocturnal species (Application 3.1; Tutorial 5).

The model described in Equation 1 is non‐linear due to the phaseshift parameters, $\left(\theta_{0}+\gamma_{i}\right)$ and $\left(\theta_{1}+\gamma_{i}\right)$. This non‐linearity makes fitting the model challenging due to a general lack of available ready‐to‐use software for fitting this type of mixed‐effect model in a frequentist framework. Instead, we can fit an equivalent model obtained by applying compound angle formulas:

(2) $$\text{logit}\left(p_{\mathrm{it}}\right)=\beta_{0}+\alpha_{1}\cos\left(\frac{2\mathrm{\pi t}}{\omega_{1}}\right)+\alpha_{2}\sin\left(\frac{2\mathrm{\pi t}}{\omega_{1}}\right)+\alpha_{3}\cos\left(\frac{2\mathrm{\pi t}}{\omega_{2}}\right)+\alpha_{4}\sin\left(\frac{2\mathrm{\pi t}}{\omega_{2}}\right)+\tau_{i}$$

where $\alpha_{1}=\beta_{1}\times \cos\left(\theta_{0}+\gamma_{i}\right)$, $\alpha_{2}=-\beta_{1}\times \sin\left(\theta_{0}+\gamma_{i}\right)$, $\alpha_{3}=\beta_{2}\times \cos\left(\theta_{1}+\gamma_{i}\right)$, and $\alpha_{4}=-\beta_{2}\times \sin\left(\theta_{1}+\gamma_{i}\right)$. Equation 2 is linear in the parameters (on the logit scale), which makes it possible to fit the model using software developed for fitting generalized linear mixed‐effect models, such as *glmmTMB* (Brooks et al., 2017) and *lme4* (Bates et al., 2015). Using Equation 2, we can fit models that include a random intercept to account for within‐site correlation and variability in frequency of site‐use (i.e. variability in $\tau_{i}$; Figure 1a). By including random coefficients for $\alpha_{1}$, $\alpha_{2}$, $\alpha_{3}$, and $\;\alpha_{4}$, we can account for variability in the timing of activity (i.e. variability in $\gamma_{i}$; Figure 1b). Further, interactions between covariates and the trigonometric terms can be used to test for hypothesized effects of biotic and abiotic covariates (e.g. season or site‐level co‐occurrence of other species) on activity patterns (Applications 3.2 and 3.3; Tutorials 6 and 7).

#### 2.2.2 Hierarchical generalized additive models with cyclic cubic splines

Similar to trigonometric models, models with cyclic cubic splines can describe periodic patterns by constraining the curves at the beginning and end (e.g. values at times 00:00 and 23:59) to match (Pedersen et al., 2019). These models belong to the generalized additive model family (Wood, 2017); as such, relationships between the response and the explanatory variables are described by smoothing criteria (usually splines) that pull parameters towards a common curve (see Pedersen et al., 2019; Wood, 2017, for a general discussion). In the context of diel activity patterns, the periodicity constraint is specified by a cyclic cubic smoother on the variable that describes the time of day within the 24‐h cycle (e.g. by hour). Models can include more than one smooth effect consisting of a sum of one of several basis functions where penalty terms control wiggliness (Pedersen et al., 2019). We can build cyclic cubic spline generalized additive models (HGAMs) with a site‐level random intercept to model variability in frequency of site‐use; by adding a smoother for the time of day within the 24‐h cycle and allowing this smoother to vary by site, we can model site‐to‐site variability in the shape of the activity curves (Application 3.2; Tutorial 6). Although some studies have modelled animal activity using cyclic cubic splines (e.g. Bischof et al., 2014; Rees et al., 2024), applications remain rare.

### 2.3 Applications

We present three empirical case studies that use hierarchical models to address common ecological questions regarding animal activity:

- Is activity concentrated in single (unimodal; e.g. diurnal, nocturnal) or multiple (bimodal; e.g. crepuscular) periods of time, or consistently maintained throughout the diel cycle (i.e. cathemeral: Tattersall, 2006; Application 3.1; Tutorial 5)?
- Are there seasonal changes in activity patterns or changes driven by environmental and anthropogenic factors (Application 3.2; Tutorial 6)?
- Does co‐occurrence lead to changes in activity (Application 3.3; Tutorial 7)?

#### 2.3.1 Data

We use camera‐trap data collected in northern Minnesota, USA, between spring 2016 and spring 2018. During each of the 5 sampling sessions (3 Springs: mid‐May to mid‐July, 2 Falls: September–October), we collected data at 100 forested sites, for a minimum of 6 weeks at each site. The methods used for collecting the data were consistent with guidelines offered by the American Society of Mammalogists. Additional details on the data collection protocol and the dataset can be found in Iannarilli et al. (2020, 2021).

#### 2.3.2 Data preparation

When using KDEs, it is strongly recommended to aggregate data into independent events to reduce short‐term correlation and improve estimates (Tutorials 9.1 and 9.3; Burton et al., 2015; Iannarilli et al., 2019; but see Peral et al., 2022). Neither of the hierarchical model approaches require this preparatory step, as records of encounters close in time will likely contribute to the same time interval. We aggregated data into independent events only when comparing hierarchical model‐based and KDE‐based estimates (Application 3.2 and Tutorial 9.4).

The trigonometric and cyclic cubic spline hierarchical models allow analysts to include information about sampling effort (i.e. information on when cameras were active, but no observation occurred). To do so, we divide the 24‐h cycle into shorter time intervals of predefined length and bin the encounter data to create a set of binary observations, *capt*_*itj*_, equal to 1 if there was at least 1 encounter at site *i* during interval *t* on day *j*, and 0 otherwise. This binning process needs to be repeated separately for each camera‐trap site during the days it was actively sampled, and the resulting encounter indicator variables will then be used as the response variable in Equation 2 (Tutorial 4). To reduce computational time, we can summarize the binary observations across the full sampling period, counting the number of successes (i.e. days with at least one encounter at site *i* during time interval *t*) and failures (i.e. days without an encounter at site *i* during time interval *t*). This allows us to model the data (aggregated to the different time intervals and sites) as a set of Binomial random variables rather than a larger set of Bernoulli random variables, one for each unique combination of site × day × time interval (Tutorial 4). If categorical covariates are included in the model, the data aggregation needs to be done separately for each of the different levels of the covariates (Tutorial 6.1). Note that this pre‐processing step would not be appropriate in cases where there is interest in modelling how activity patterns change as a function of covariates that vary within the sampling period (e.g. Julian day: Iannarilli et al., 2021).

In the examples that follow, we binned the data using 24 1‐h intervals, but users can select any shorter or longer length depending on the desired temporal resolution and the computational resources available. It is challenging to provide general rules regarding the appropriate time‐interval length for modelling and the number of detections (or detections per site) needed in order for the models to perform well. In general, shorter time‐interval lengths result in longer computation times and might lead to model convergence issues when data are sparse. Also, a common suggestion is to have 10 or more clusters before applying hierarchical models with random effects, with more sites needed when detection probabilities are low or variability amongst sites is high (Kéry & Royle, 2015). In general, we recommend using simulations to help determine an appropriate sampling and analysis plan. Data preparation is described in detail in Tutorial 4.

#### 2.3.3 R‐packages

We fit trigonometric hierarchical models using the *mixed_model* function in *GLMMadaptive* (Rizopoulos, 2020); for these models, mean estimates and their 95% confidence intervals are obtained using *effectPlotData* in *GLMMadaptive* (Rizopoulos, 2020) and then back‐transformed from the logit to the probability scale using the function *plogis* in base R. The same models can be fit using other R‐packages (Table 2); we discuss pros and cons of the different packages in the Discussion section. We fit cyclic cubic spline hierarchical models using the function *bam* in package *mgcv* (Wood, 2017) and *predict.bam* to calculate point estimates and 95% confidence intervals based on these models. All the examples are executed in the R programming language (R Core Team, 2022). Data, code, and Tutorials are stored as a static copy at the U.S. Geological Survey (Iannarilli et al., 2024a) and at the Data Repository for the University of Minnesota (Iannarilli et al., 2024b). The material is also available in GitHub at https://github.com/FabiolaIannarilli/HMs_Activity and an HTML version of the Tutorials could be browsed at https://hms‐activity.netlify.app.

**TABLE 2:** Syntax to fit hierarchical models using alternative R packages: *GLMMadaptive* (Rizopoulos, 2020), *glmmTMB* (Brooks et al., 2017), and *lme4* (Bates et al., 2015) for the trigonometric GLMMs and *mgcv* (Wood, 2017) for cyclic HGAMs. *cos*(*Time*) is a placeholder for a series of sine and cosine terms (see code in Section 2.3.4).

	Random intercept‐only	Random intercept and slope	
		Uncorrelated random effects^b^	Correlated random effects
*GLMMadaptive*	fixed = cos(Time), random = ~ 1|Site	fixed = cos(Time), random = ~ cos(Time)||Site	fixed = cos(Time), random = ~ cos(Time)|Site
*glmmTMB* and *lme4* ^a^	∼ … + (1|Site)	~ … + (1|Site) + (0+ cos(Time)|Site)	~ … + (cos(Time)|Site), which is equivalent to ~ … + (1+ cos(Time)|Site)
*mgcv*	~ … + s(Time, bs = “cc”, k = 12) + s(Site, bs = “re”),	~ … + s(Time, bs = "cc”, k = 12) + s(Time, bs = “cc”, k = 12, by = Site) + s(Site, bs=“re”)	NA

^a^ Adapted from https://bbolker.github.io/mixedmodels‐misc/glmmFAQ.html#model‐definition.

^b^ Covariance matrix is assumed to be diagonal.

#### 2.3.4 Evaluating hypotheses regarding the shape of activity curves

In this first example, we illustrate how trigonometric hierarchical models can be used to assess whether a species' activity pattern can be classified as unimodal, bimodal, or cathemeral (Aschoff, 1966; Tattersall, 2006). We fit and compare three candidate models describing these alternative options using records of coyotes (*Canis latrans*; Tutorial 5). The first model is a null model that assumes activity exhibits no diel variation (i.e. cathemeral activity pattern):null_mod <- mixed_model(fixed = cbind(success, failure) ~ 1, random = ~ 1 | Site, family = binomial(), data = occasions_cbind )


We then code models for unimodal and bimodal patterns following Equation 2:unimodal <- mixed_model(fixed = cbind(success, failure) ~ cos(2*pi*Time/24) + sin(2*pi*Time/24), random = ~ cos(2*pi*Time/24) + sin(2*pi*Time/24) || Site, family = binomial(), data = occasions_cbind )bimodal <- mixed_model(fixed = cbind(success, failure) ~ cos(2*pi*Time/24) + sin(2*pi*Time/24) + cos(2*pi*Time/12) + sin(2*pi*Time/12), random = ~ cos(2*pi*Time/24) + sin(2*pi*Time/24) + cos(2*pi*Time/12) + sin(2*pi*Time/12) || Site, family = binomial(), data = occasions_cbind )


All three models contain a random intercept for *Site* and, thus, accommodate site‐level variability in the frequency of site‐use. The unimodal and bimodal models include random slopes and, thus, also accommodate site‐level variability in the timing of activity. Figure 2 reports the activity patterns predicted by each model. We can compare model support using either the Akaike Information Criterion (AIC; Burnham & Anderson, 2002) or a Likelihood Ratio Test (LRT). Based on AIC, the model describing a bimodal pattern is the most supported (i.e. it has the lowest AIC value):

AIC(null_mod, unimodal, bimodal) df AIC deltaAICbimodal 6 1311.965 0.000unimodal 4 1317.350 5.385null_mod 2 1365.230 53.265



A LRT run using the code:lmtest::lrtest(null_mod, unimodal, bimodal)


returns similar results (unimodal vs. bimodal: *p*‐value = 0.02; unimodal vs. null model: *p*‐value > 0.001).

#### 2.3.5 Modelling activity patterns with covariates

We now illustrate how hierarchical models can be used to quantify seasonal differences in American black bear (*Ursus americanus*) activity patterns between Fall (September–October; *Season* = 0) and Spring (mid‐May to mid‐July; *Season* = 1; Tutorial 6.1). For an example assessing the effect of a continuous covariate, see Tutorial 6.2. To explore the covariate effect, we summarized the number of encounters at each site and time interval (i.e. hour of day) across the two seasons. Here, we focus on illustrating cyclic cubic spline hierarchical models, but we also compare the results to those obtained using trigonometric hierarchical models and KDEs (Tutorials 6.1 and 9.4).

We present two cyclic cubic spline HGAM structures to model activity patterns when including a covariate effect. The first structure is analogous to a random intercept‐only generalized linear mixed model (GLMM) and only accounts for variability in the frequency of site‐use:mod_cycl1 <- bam(cbind(success, failure) ~ Season + s(Time, bs = "cc", k = 12, by = Season) + s(Site, bs = "re"), knots = list(Time = c(0,23)), family = "binomial", data = occasions_cbind )


We include a fixed linear term for *Season*, and a smoother s(Time, bs = “cc”, k = 12, by = Season) for *Time* that is allowed to vary by *Season*. For this smoother, we set the number of basis functions, *K*, to 12; estimates are typically robust to this choice as long as *K* is sufficiently large (Wood, 2017, p. 243). The argument bs = “cc” specifies a cyclic cubic spline as the smoother for *Time* and guarantees that predicted end points of the estimates match at the locations given by the knots argument. We specify a random effect smoother bs = “re” for the covariate *Site*.

**FIGURE 3:**
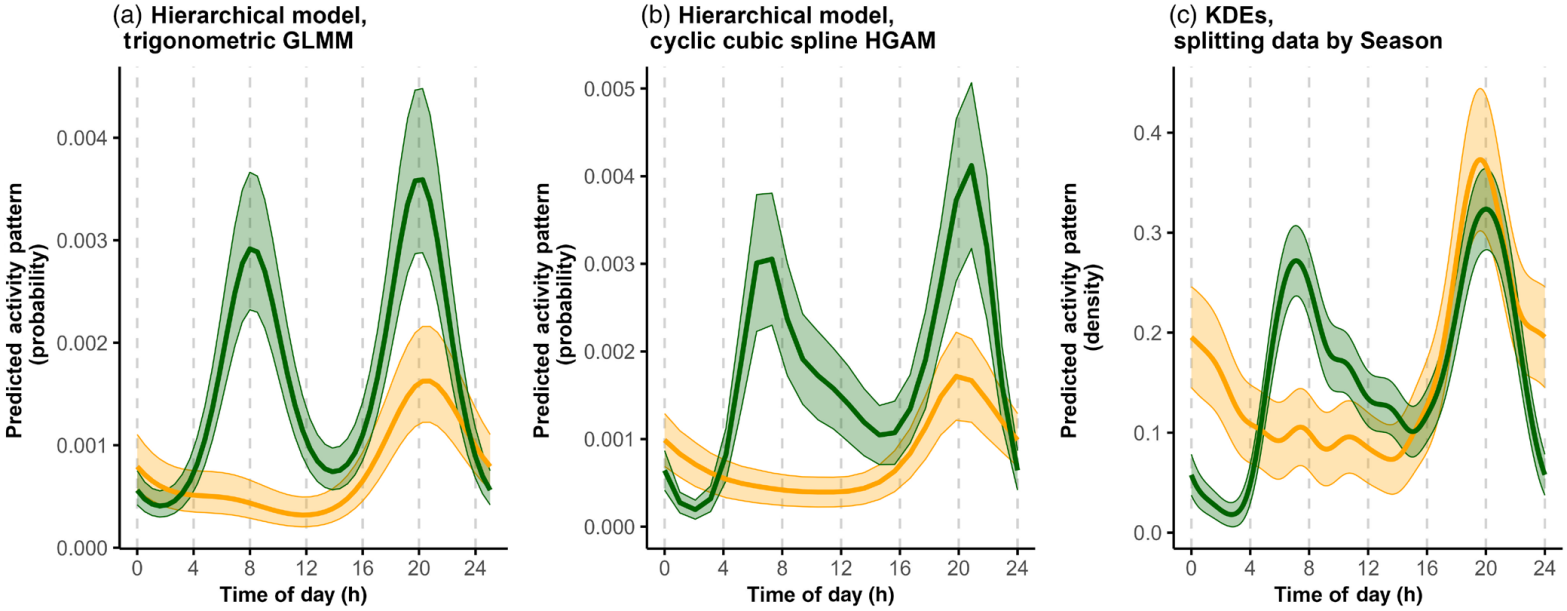
Black bear (*Ursus americanus*) mean activity patterns in Spring (green) and Fall (orange) were estimated from camera‐trap data collected in northern Minnesota between 2016 and 2018. To facilitate comparison across models, records were aggregated across independent encounter events using a 30‐min threshold (Tutorial 9.4). Panels (a and b) report estimates of conditional means from hierarchical models, (a) using a (random intercept‐only) trigonometric GLMM and (b) a cyclic cubic spline HGAM. Panel (c) reports estimates of marginal means using KDEs (after splitting the data based on *Season*). Lines and shaded areas represent point estimates and 95% confidence intervals for each method. For KDEs, confidence intervals were estimated via bootstrapping.

In Figure 3, we compare the HGAM estimate to estimates from a trigonometric (random intercept‐only) GLMM and KDEs (Tutorial 9.4). All methods identify large differences in seasonal activity patterns, with a switch from a clear bimodal crepuscular activity pattern in the Spring (peaks at 7:00 and 20:00) to a unimodal activity pattern in the Fall (peaks around 20:00; Figure 3). Because the start of the Fall sampling coincided with the beginning of the bear hunting season in Minnesota, we suspect the decline in activity around sunrise is linked to high levels of hunting activities during the morning. Importantly, although hierarchical models and KDEs estimate similar patterns for each season, we stress that KDEs only allow for comparisons of relative activity levels within a curve, and not amongst curves. By accounting for variability in sampling effort, trigonometric and cyclic cubic spline hierarchical models allow researchers to directly interpret the y‐axis in the activity plots (e.g. Figure 3a,b) as the probability of activity. Thus, although all methods allow us to conclude that bears had a peak in diel activity patterns around 20:00 in both Spring and Fall, only the hierarchical models allow us to conclude that the probability of activity at 20:00 was higher in the Spring than in the Fall (Figure 3). We can also use hypothesis tests (trigonometric GLMM: *p‐values* for all the interaction terms between sin/cos and the variable *Season* < 0.001; cyclic cubic spline HGAM: *p*‐values of the two levels of the smoother for *Time* by *Season* < 0.001) or AIC (ΔAIC trigonometric GLMM null model versus model with *Season* = 170.95; ΔAIC HGAM null model versus model with *Season* = 475.28) to compare models with and without the covariate of interest. Additionally, we can conclude that there is a relatively high level of amongst‐site variability in the frequency of site‐use (trigonometric GLMM: $\hat{\sigma_{\tau }}=0.88$). Lastly, we note that the KDE activity curves tend to be wigglier than the hierarchical model estimates, likely due to the assumption of independence amongst observations when calculating an appropriate smoothing parameter. This effect can be particularly strong when data are sparse.

We can further extend the HGAM structure above to also explore variability in the timing of activity (Tutorial 6.1.2) by adding two additional components: a smoother for *Time* that depends on *Site*, which allows the estimated activity curves to vary by *Site*, and a global smoother for *Time*, that shrinks the site‐specific activity curves towards a general mean curve:mod_cycl2 <- bam(cbind(success, failure) ~ Season + s(Time, bs = "cc", k = 12) + # global smoother s(Time, bs = "cc", k = 12, by = Site) + s(Time, bs = "cc", k = 12, by = Season) + s(Site, bs="re"), knots = list(Time = c(0,23)), family = "binomial", data = occasions_cbind )


**FIGURE 4:**
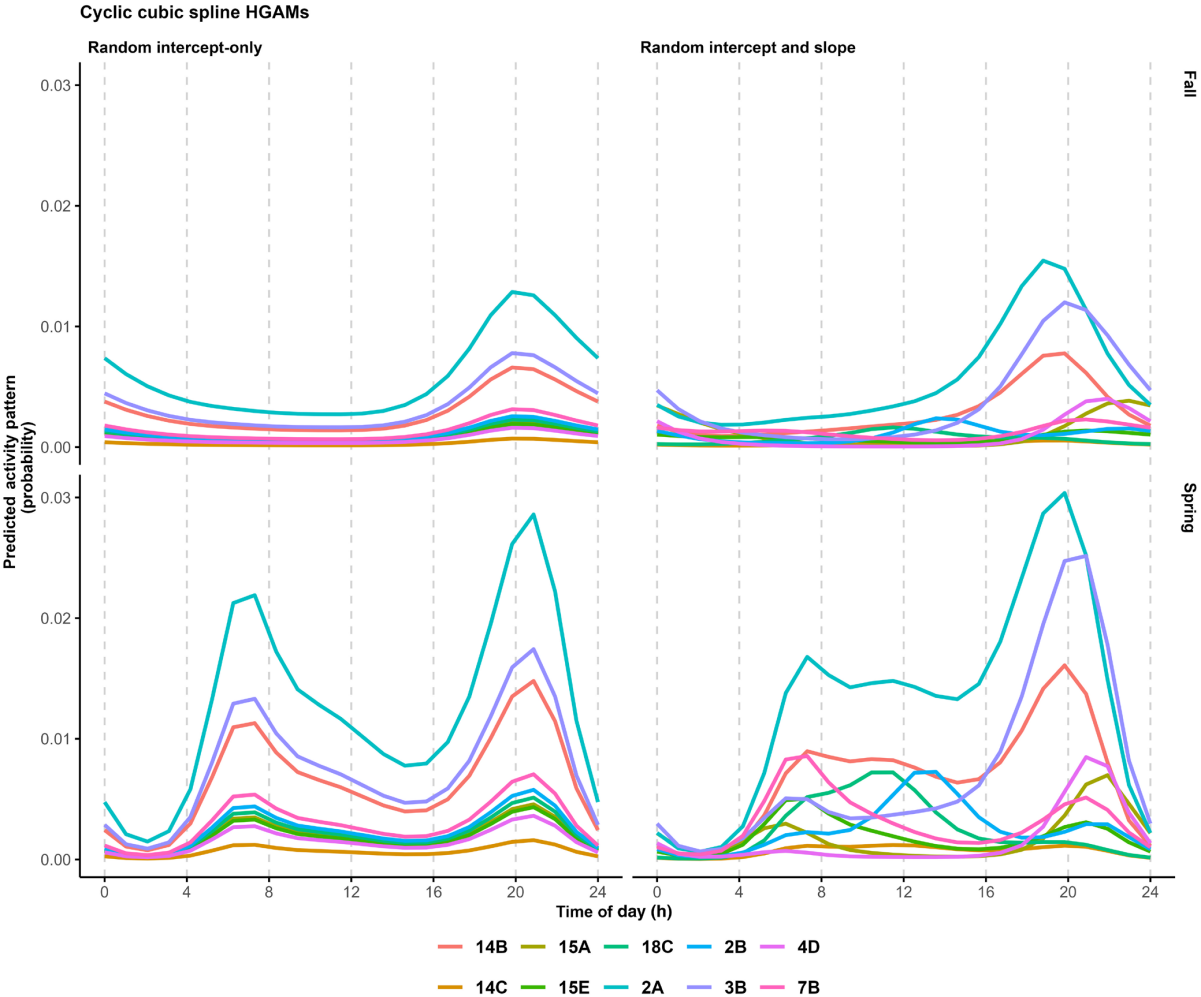
Estimated site‐specific activity patterns of American black bear (*Ursus americanus*; showing 10 of 100 sites) using random intercept‐only (left panels) and random smooth (right panels) HGAMs in Spring (bottom row) and Fall (top row).

This structure resembles a random intercept and random slope GLMM and better describes our data compared with the previous HGAM structure (ΔAIC = 8.39). Estimates of site‐specific activity curves (Figure 4) highlight considerable site‐to‐site heterogeneity, an aspect that has been largely ignored in studies of diel activity patterns.

#### 2.3.6 Temporal partitioning

Hierarchical models can also be used to quantify differences in activity patterns at sites with and without competitors. We explore whether coyote activity patterns differ at sites where grey wolves (*C. lupus*) were and were not observed to see if temporal partitioning may offer a mechanism that facilitates co‐existence (Tutorial 7). Here, we report marginal mean activity patterns since we are interested in differences between groups of sites (sites where the competitor is either present or absent).

**FIGURE 5:**
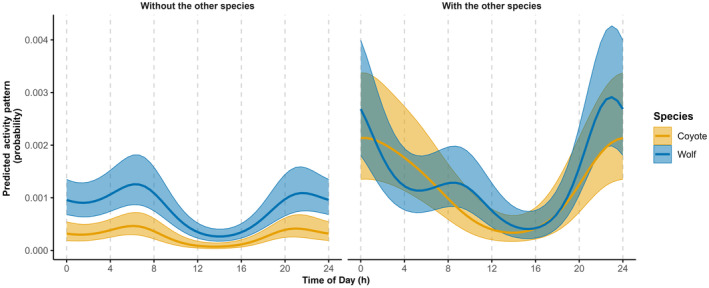
Marginal mean activity patterns of coyotes (*Canis latrans*) and grey wolves (*C. lupus*) when the other species was or was not photographed at least once at the same site during a specific session; estimates are based on random intercept‐only trigonometric GLMMs (Tutorial 7). Data were collected in northern Minnesota in 2016–2018 using camera traps. Lines and shaded areas represent point estimates and 95% confidence intervals.

We found that wolves and coyotes exhibited higher activity levels when they were detected at the same sites, suggesting that some site features or the presence of the competitor might lead to higher activity levels (Figure 5). Though wolves were more active than coyotes, both species also exhibited similar temporal patterns when found alone, with activity mostly at night (between 19:00 and midnight) and in the early morning (between 4:00 and 8:00; Figure 5). When the two species co‐occurred, coyotes shifted towards an unimodal pattern with most activity between 23:00 and 4:00, and wolf activity peaked earlier in the night (between 21:00 and 1:00; Figure 5). These differences might also be attributable to spatial factors not considered, such as variation in population density, prey diversity or density, or in levels of human disturbance at sites where the species co‐occurred or not (Frey et al., 2020).

## 3 CAVEATS

In this How‐to guide, we illustrate how to pair trigonometric and cyclic cubic spline models with a hierarchical modelling approach to estimate activity patterns from time‐stamped data. This method leverages well‐established statistical frameworks and offers several advantages. Like KDEs, the trigonometric or cyclic cubic spline component of these models accommodates circular data. However, unlike KDEs, a hierarchical modelling approach can simultaneously account for multiple sources and types of variability (e.g. unmodeled site‐to‐site variability, unequal sampling effort; Table 1) whilst enabling analysts to assess the effects of biotic and abiotic factors in a cohesive modelling framework. By accommodating the periodicities inherent to many species' activity patterns and non‐independence of observations, these hierarchical models are well suited for analysing time‐stamped data from camera traps and other similar static sensors (e.g. audio recorders). Most importantly, trigonometric and cyclic cubic spline hierarchical models provide a robust framework to test hypotheses regarding the effects and relative importance of both categorical and continuous variables on diel activity patterns with widely used model selection tools (e.g. AIC, likelihood ratio tests).

The choice between trigonometric and cyclic cubic spline hierarchical models may depend on a user's familiarity with these two statistical methods. However, only trigonometric hierarchical models can impose specific model structures to test hypotheses regarding the shape of diel activity patterns (Application 3.1). We chose *GLMMadaptive* (Rizopoulos, 2020) amongst the many packages available for fitting GLMMs because it directly provides estimates of both conditional and marginal means. For users interested in only estimating conditional means, other packages can also be used (Table 2) and might provide reduced computational time. Additionally, marginal means can still be estimated when using these packages either via simulation‐based approaches or by leveraging R packages such as *ggeffects* (Lüdecke, 2018) and *marginaleffects* (Arel‐Bundock, 2024). Another consideration is that HGAMs use the data to determine an appropriate level of smoothing, which has both pros (the user does not need to decide this on their own and the level of smoothing should effectively navigate the bias/variance tradeoff inherent to smoothing) and cons (inference becomes more challenging due to using the same data to determine an appropriate level of smoothing and for inference).

In the frequentist framework, random intercepts are straightforward to include and allow analysts to account for variability in the frequency of use and repeated observations at the same site. Accounting for shifts in the timing of activity is more difficult because adding a random slope often results in a more challenging optimization problem and considerably longer computational times, especially when analysing large datasets. Ecologists commonly fit models containing only random intercepts and not random slopes (Muff et al., 2020). Although this approach can result in biased estimators when the effects of covariates (and here, the timing of activity) vary by sites, we expect this approach will typically provide a better description of activity patterns than KDEs that ignore all site‐to‐site variability. When possible, models that allow for site‐to‐site variability in both the frequency and timing of use should be considered. Alternatively, these models could be fit using the Bayesian approach, which is particularly apt at accommodating complex model structures, including non‐linear hierarchical models (Tutorial 10.3).

Some caution is warranted when using common model selection procedures, such as AIC, for hierarchical (i.e. mixed‐effect) models (Bolker et al., 2009). Users must also choose between marginal and conditional versions of the AIC depending on whether they are most interested in inference at the cluster (i.e. site) or population level (Greven & Kneib, 2010). We used marginal AIC in our comparisons as this is the default when using the AIC function in R. The *cAIC4* package (Säfken et al., 2021) has an implementation of a conditional AIC for *lme4* and *mgcv* packages, but not for *GLMMadaptive*. Pederson and colleagues (2019) discourage the use of (Generalized) LRT for HGAM model comparisons due to the lack of sufficient theory, and Wood (2017) notes that *p‐values* tend to be too small (usually half of what they should be) because HGAMs use the data twice (once to choose an appropriate level of smoothing and then secondarily for inference). This issue might be less relevant when reported *p*‐values are extremely small (<0.0001 as in some of our examples), but it becomes crucial when they are higher than 0.02 (assuming a significance level of 0.05). AIC‐based comparisons of HGAMs with and without a global smoother (as in the case of the two structures presented here) are also not recommended because AIC will tend to favour the structure without the global smoother (Pedersen et al., 2019). In general, we emphasize that model selection should instead primarily be based on careful consideration of the study system, the experimental design, and the research questions of interest (Fieberg & Johnson, 2015).

Whilst we only present a selection of model structures and procedures, further extensions are possible. For example, the hierarchical model framework can be adapted to include spatiotemporal random effects (e.g. via Gaussian processes) to account for additional sources of variability (e.g. spatially varying population densities), and models may be formulated using a Poisson distribution (instead of a binomial), which might provide computational advantages (Tutorial 10.2). Additionally, multiple covariates could be included in the same model. Although we do not present it here, the code provided can be extended to accommodate procedures that correct for different sunrise and sunset timing during the sampling period (Vazquez et al., 2019) and the temporal intervals can also be reported in radians (as required by the three R packages that use KDEs to estimate activity curves; Tutorial 9.2).

## 4 CONCLUSIONS

In a world with increasing human‐driven pressures on wildlife and ecological communities, there is a need for methods that can explore and quantify the combined effects of natural and anthropogenic factors on animal behaviour (Berger‐Tal et al., 2011; Frey et al., 2017). Hierarchical models fitted with trigonometric and cyclic cubic splines address this need for activity patterns. Combined with purposely designed experimental studies (Smith et al., 2020) and the ongoing technological advancements of devices and AI innovations in processing and extracting information from the data collected, these methods can increase our understanding of interspecies relationships in natural and human‐modified environments and better inform conservation and management decisions.

## AUTHOR CONTRIBUTIONS

John R. Fieberg and Fabiola Iannarilli conceived the ideas and designed the methodology; John Erb and Fabiola Iannarilli collected the data; Fabiola Iannarilli, John R. Fieberg, and Brian D. Gerber developed and validated R code and tutorials; Fabiola Iannarilli wrote the original draft and prepared the visualizations; All authors contributed critically to the drafts and gave final approval for publication.

## CONFLICT OF INTEREST STATEMENT

The authors declare no conflict of interest.

## Data Availability

Data, R code, and Tutorial are stored as a static copy in the Data Repository for the University of Minnesota (DRUM) at https://doi.org/10.13020/w1x9‐3650 and at the U.S. Geological Survey at https://doi.org/10.5066/P13UTDBG; the material is also available as a living repository in GitHub (https://github.com/FabiolaIannarilli/HMs_Activity). An HTML version of the tutorial could be browsed at https://hms‐activity.netlify.app.
